# Scrofula; Its Nature, Its Causes, Its Prevalence, and the Principles of Treatment

**Published:** 1846-07

**Authors:** 


### ART. XI.

*Scrofula; its Nature, its Causes, its Prevalence, and the Principles of Treatment.* By BENJAMIN PHILLIPS, F.R.S., Assistant Surgeon to the Westminster Hospital.—*London,* 1846. 8vo, pp. 379.

THIS is one of the few books which may be said to be after the critic's own heart. It is a book that was wanted; the object of it is excellent; it has been carefully planned; the investigations required by the plan have been conducted with the utmost energy, industry, and carefulness, in a sufficiently extensive field, and over a sufficiently long period of time; the immense mass of materials thus obtained has been examined and weighed with the greatest attention and impartiality; the inferences and results have been honestly and cautiously deduced and elaborated, and condensed into the smallest possible space consistent with perspicuity; while the whole style and manner of the composition is such as ought to characterize the production of a well-educated, a learned, and an experienced surgeon. In saying thus much, we are far from implying that the work is a perfect one; and we suspect that the author is himself the last person who would claim such a character for it. It is, however, but doing simple justice to Mr. Phillips to state, that we believe the work to be as complete as it could well be made under all the circumstances of the case. Though generally agreeing with the author in his views and conclusions, we occasionally differ with him in both; and in presenting to our readers as fair and complete an analysis of his book, as we can, we shall not hesitate to display what we may regard as its errors with the same freedom as we hold up its manifold excellences to the admiration and imitation of his brethren. It would be paying the author of such a book a compliment most unworthy his acceptance, to hesitate to express our honest and candid opinion on any of the questions discussed in it, whether that opinion was in unison with his own or in opposition to it. His object, like ours, is to reach the truth; and he will not, we are sure, quarrel with us or any one else who can aid him, even in the least degree, in attaining it.

We cannot do better than introduce our review of this work in the words of its author.

"In the course of my professional practice, and especially in the discharge of the duties entrusted to me as surgeon to a large metropolitan infirmary, the treatment of scrofulous affections formed a subject of frequent and anxious consideration. I often felt desirous to relieve myself of anxiety, by consulting the experience of others, as recorded in their published works, but I found that something was wanting in all those to which I referred—too much was assumed, too little carefully examined; and although many of the works on scrofula which we possess have deservedly acquired a high reputation, at an early stage of my investigations I was led to the conclusion, that much was yet wanting to complete our knowledge of the disease . . . . . Fully satisfied that a more extended collection of facts, and a more accurate classification of phenomena than had yet been attempted, were necessary and practicable, I entered upon the task which has now been brought to a close, and men who have been engaged in such extensive and minute inquiries can alone appreciate the patient labour which has been devoted to the work.

"The materials which I have obtained and used, are probably the most ample that have ever been collected and brought to bear on a single medical subject; and if I had contemplated the difficulties I have encountered in procuring information, or the extent to which the inquiry would have proceeded, I should probably have been deterred from prosecuting it to a conclusion. But as is the case in all such undertakings, the obstacles and discouragements were only gradually unfolded, and the labour was lightened by the interest which the subject itself excited." (p. 4.)

Mr. Phillips commenced his more extended collection of facts with his own work-circle at the Marylebone Infirmary. Finding this too limited, he visited, to use his own words, a large number of the national schools, the parochial establishments, and the charitable institutions which are found in every part of the metropolis. He was thus enabled to determine whether the healthier and wealthier localities of St. George's, Hanover square, St. Marylebone, and Kensington, were more lightly visited by the disease than the comparatively poor and unhealthy districts of Shoreditch, Bethnal Green, St. Giles's, and the lower part of Westminster. Having ascertained, as far as was practicable, the condition of the population of the metropolitan districts, where the effects of close packing may be expected to be keenly felt, he pursued the inquiry through districts where the population was widely spread; and he was thus enabled to compare the condition of the people living under very different circumstances, and to ascertain the influence of crowding upon human life, in so far as concerns scrofula. He extended the investigation to the mining and factory districts, with a view to determine how far particular occupations tended to develop the disease. He caused children to be examined in the north and the south, the east and the west of our own country, so as to be able to estimate the influence of particular localities in our own island to determine the affection; and he extended his researches throughout Ireland and Scotland, so as to be enabled to estimate the influence of particular articles of food; and the correctness of the evidence so obtained, has been to a certain extent tested by the condition of recruits, and of persons committed to prisons; by the mortuary tables of the Registrar-General, so ably analysed and arranged by Mr. Farr; by those of the Irish census, so well digested by Mr. Wilde—in so far as they could be made available in an inquiry like the present; and by a large number of hospital and dispensary returns.

Mr. Phillips's ambition grew by what it fed on, and he now found that the sphere of the British isles formed too limited a boundary for observation. He therefore pushed his statistical inquiries into the four great continents. Through the kindness of Lord Aberdeen, he has obtained most valuable reports from Russia, Austria, Prussia, Bavaria, Portugal, and Holland. He has acquired important information from the northernmost portions of Europe, as well as from France and Belgium, and in Bavaria, Belgium, Tyrol, and Switzerland, he has been enabled himself to verify the accuracy of the information with which he had been furnished. He has also obtained returns of the condition of children in so much of the United States of America as is found on its eastern shores, between Boston and New Orleans; from a certain portion of the Bengal presidency; from some districts in China; and from portions of Egypt, Syria, and Greece, abutting on the Mediterranean Sea, and from Madeira.

Mr. Phillips drew up forms of inquiry which he procured to be transmitted to all such persons as were likely to assist in his researches.

Early in his progress Mr. Phillips found the definition of scrofula and of the scrofulous temperament to be rather a knotty difficulty. He was very much in the predicament of Richard Wiseman, who had to select the persons to be placed before Charles the Second, to be touched for "the evil," and he came to a conclusion very similar to that at which Wiseman arrived. Wiseman observed that none of the definitions which limited struma to an enlargement of the cervical or inguinal glands, sufficiently expressed the disease termed in England the king's evil. "For I appeal," he says, "to the practitioners of this nation, whether physicians or chirurgeons, desiring them to say whether there be not many tumours and ulcers commonly judged to be the evil, which are contained in no cystis at all, neither do they concrete into a glandule. I instance in the upper lips chopt, divers tumours by congestion in the musculous parts of the body, others in the bones, &c.; so that either we must exclude all these from the king's touch, or alter our definition." In his diagnostics for the court, he, however, takes the enlarged cervical glands for a pathognomonic sign. He says, "These which we present to his majesty are chiefly such as have this sort of tumour about the musculus mastoides, or neck, with whatever other circumstances they are accompanied; nor are we difficult in admitting the thick-chapped upper lips and eyes affected with a lippitudo; in other cases we give our judgment warily." Mr. Phillips had to define cases of scrofula for statistical tables, and not for "the king's touch." He limited the pathognonomic signs to enlarged cervical glands discoverable by the touch or the sight; sinuses, or ulcerations resulting from them; and ordinary scrofulous bones or joints. We subjoin the form which Mr. Phillips employed for the collection of the extensive statistical data to be subsequently noticed, together with his explanatory remarks:

| | | | |
|---|---|---|---|
| | BOYS. | | |
| 1. Number of children examined between 6 and 16 years. | 2 Number of such children who have decidedly fair hair, and light blue or light gray eyes, and a fair, soft skin. | 3 Number of children exhibiting any of the following marks of scrofula: enlarged cervical glands discoverable by the touch; sinuses or ulcers, succeeding to such glands; scrofulous bones or joints, or the consequences of them. | 4. Number exhibiting the evidences of scrofula described in the third column, and possessing the characters described in the second column. |
| | GIRLS. | | |
| | | | |
| | DIET. | | |
| | | | |

"The object of the second question is to ascertain how large a proportion of cases is found among those children who have what may be fairly called a light complexion. With reference to the third question, it is not pretended that every mark of scrofula is included; but as opinions as to what is and what is not scrofula are very different, it seemed important to fix upon certain points which did not admit of much controversy. In a healthy child, the lymphatic glands about the jaw and the neck are not perceptible on passing the finger over them; whenever, therefore, they can be detected, they are to that extent in an abnormal condition, though not necessarily scrofulous. All that is required for the purposes of the present inquiry is to pass the fingers along the spaces under the lower jaw, and parallel to and in front of the mastoid muscle; and if sensible enlargements can be detected, the case would be enumerated in the third column, which includes all cases in which such enlargement can be perceived." (p. 10.)

The mere signs of scrofula do not teach us much as to its nature. To this question Mr. Phillips devotes the second chapter of his book, previously appropriating a chapter to the exposition, examination, and demolition of antecedent theories. He plunges at once into the middle of his epic; thus:

"I conceive, then, that scrofula is a disease of the constitution, and that it is most clearly manifested by certain external signs, of which swelling of the subcutaneous lymphatic ganglia is the most conclusive. But *tumid glands*, however, wherever they may be situated, are not always a proof that a constitution is scrofulous; they may be the result of local irritation, in an apparently untainted constitution. The glands in the groin may swell from a sore on the foot; a mesenteric gland may swell under the influence of an ulcer in the intestine; a cervical gland may enlarge under the irritation of teething, or of scalp disease. A *tumid gland*, even *in the neck*, is then no proof that the constitution of the individual in whom it is found is scrofulous. But supposing one, or several cervical glands to become tumid, in the apparent absence of any obvious local irritation, this would constitute a strong ground for suspicion that the constitution was suffering under the taint of scrofula. It would not, however, amount to more than suspicion, and the suspicion could scarcely receive absolute confirmation, unless we have the oppportunity of observing the contents of the tumour itself. Unless the swelling of the gland be accompanied by the deposit of a product, hereafter to be described, known as *scrofulous matter*, the proof of a scrofulous constitution is, in my judgment, wanting.

"In so far then as the local manifestation is concerned, the question, "What is scrofula?" admits of a tolerable satisfactory solution; but as I regard the local affection, wherever seated, as clear evidence of constitutional disease, it is most important to inquire what that state of the constitution is which causes the local affection; and whether, in the absence of the local affection, there be any certain means of determining whether a constitution be scrofulous or not. Because it may be that the means at our disposal may be powerless to remove the matter when once deposited." (p. 27.)

Mr. Phillips is well aware of the objection that may be advanced against this view of the case. There are scrofulous diseases in which no scrofulous matter can be demonstrated; scrofulous ophthalmia, for instance.

"Supposing such an objection to be made, my answer is this: that the ophthalmia in these cases is a simple catarrhal inflammation, set up in a scrofulous constitution, and acquiring its particular characters, not in virtue of anything specific in its nature, but in consequence of the state of the constitution upon which it is grafted. The particular character to which I allude is a peculiar irritability, which is a prominent feature in inflammation set up in debilitated constitutions, and there is certainly nothing uncommon in such inflammation in persons whose health has been broken by other causes than scrofulous disease. At the time I visited the Whitechapel workhouse, 'scrofulous ophthalmia' was epidemic among the girls. Of eighty-nine present, seventy were suffering from the disease; but of the eighty-nine, only fourteen exhibited any of the ordinary marks of scrofula; and the proportion of girls who presented marks of scrofula, found among those suffering from ophthalmia, was not greater than among those who were free from it.

"I believe that diseases regarded as scrofulous, but in which no scrofulous matter is present, are not scrofulous at all, but simply the result of such low inflammatory action as is often set up in a debilitated state of the constitution." (p. 28)

We think this answer to the objections is inconclusive, for sundry reasons. Firstly, we are told nothing of that "peculiar irritability," which is the pivot of the answer. Secondly, we doubt very much whether all the cases of so-called scrofulous ophthalmia, noted in the Whitechapel workhouse, were true examples of that disease; that is to say, were genuine examples of that well-known variety of ophthalmia, which occurs, from time to time, among strumous children, and with the character of which none is better acquainted than Mr. Phillips. Did it not rather come into the category of those epidemic ophthalmias which, in their more formidable forms, have desolated barracks and prison-houses, more especially on the Continent, and have been made the subject of so much discussion, and the cause of so much authorship? We think it beyond question that such ophthalmia prevails occasionally in a mild and modified form amongst the pauper children maintained in the workhouses. Thirdly, it is certain that there may be extensive scrofulous disease of the joints (one of Mr. Phillips's pathognomonic signs, and one of those on which his statistics are founded), and yet the cervical glands be unaffected. In such cases we must adopt Mr. Phillips's own conclusion, that "those diseases regarded as scrofulous, but in which no scrofulous matter is present, are not scrofulous at all." "The consequences of scrofulous bones, or joints," may also exist without any trace of scrofulous matter; so that, after all, we fall back to Wiseman's principle of selection for his majesty's touch.

*The signs of a scrofulous constitution,* as generally stated by authors, are fairly open to the objections advanced by Mr. Phillips. They are too indefinite; in a large proportion of cases they are not present; in another large proportion they exist, but without signs of scrofula. The following is Mr. Phillips's account—and it is a masterly one—of the qualities most commonly met with in those who are tainted with the disease:

"In the form of the body there is usually observable a want of muscular development; but even this is often absent. There is often an appearance of plumpness, or roundness, which is the result not of muscular development, but simply of an hypertrophied, or infiltrated condition of the cellular tissue, and which rapidly disappears under fatiguing exercise, privation, or disease. Commonly there is a general paleness and coldness of the surface of the body, which is owing to a feeble circulating apparatus; but in a large number of cases, about one fifth of the whole, that paleness does not extend to the face. The colour of the hair is very variable, but for the most part it inclines to a dark tint. Of nearly nine thousand scrofulous children I have myself examined, a little over thirty-two per cent. had light hair and eyes. The alæ nasi may be broad, but for the most part they are not so. There is not, as some persons have supposed, anything constant in the shape of the lower jaw, or in the appearance of the teeth. The abdomen is commonly tumid. The whole of the mucous surfaces are especially liable to derangement; discharges from the nose, the eye, and the ear are common. The digestive mucous membrane affords early indications of suffering; the tongue has commonly a dirty whitish coating; the tonsils are usually enlarged, and they are often so tumid as to impress a disagreeable and frequently husky character upon the voice, and to cause snoring when the patient is asleep. A still more deleterious influence is exercised by these tumid bodies; they lessen so much the channel for the passage of the air in respiration, that the sufficient development of the chest may be interfered with. The stomach and bowels are frequently disordered, and digestion is ill performed; acrid eructations are common; flatulence is often very troublesome; and the action of the bowels is very irregular, sometimes relaxed, at others constipated. Sometimes the evacuations are clay-coloured, very offensive, and of varying consistency; at others having a redundancy of bile. Similar evidences of derangement are observed in the air-passages, commencing at the nose (which exhibits increased secretions upon the occurrence of very slight variations in the temperature), and passing through their whole length. Similar phenomena are observed in the mucous tissue of the genito-urinary system; the bladder often shows an impatience of the presence of the urine, and the desire to void it is often frequent. The skin, though often dry and hard, is sometimes the seat of a considerable greasy exhalation; sometimes it is found to be fetid and sour. The acidity of the exhalation may be so decided as to determine a reaction upon litmus paper. In many of the cases observed by Mr. Kaye, on the Mediterranean coast, it was so. The scalp and other parts of the cutaneous integuments are often the seat of eruptive affections. The absence of vascular and muscular energy often causes the child to lie and sit about much, and indisposes him to enter into the energetic games of his playfellows. As to the intellectual development claimed for scrofulous persons, I am bound to say that it is usually wanting. That many scrofulous children present that character is quite true; but the result of very careful observation has convinced me that the overwhelming majority are without those superior intellectual qualities which have been pointed out as their ordinary character. Among the better classes the feebleness of a scrofulous child attaches to him an interest which, without it, he might not have enjoyed. To compensate for his physical inferiority, the anxious parent seeks to make him mentally superior to his bodily stronger fellows, and frequently succeeds; but often the limit of healthy action is passed, the nervous and intellectual systems have the vital action concentrated on them too intensely. The sufferer loses flesh, the general health languishes, and the intellectual faculties may give way, destroyed by an opposite, but not less sure, method than that which breaks down the poor man's child." (pp. 30-2.)

We have not yet got to the author's ideas as to the nature of scrofula: we have the pathognomonic signs, and we have the constitutional signs. But the scrofulous constitution is not scrofula; there is a wide divergence from the state of health—the constitution is fitted for the development of scrofula—but the scrofulous constitution is only a reality when the deposit occurs. Mr. Phillips observes:

"That condition of the economy, then, which is favorable to the formation of scrofulous matter is not scrofula, but a diathesis, or disposition; it may exist long, it may cause the tumefaction of many glands, but, under favorable circumstances, it may disappear without the deposit of a single particle of that product, whose presence in the glandular structures is, I conceive, necessary to constitute scrofula Again, I say, the condition of the system favorable to this deposit is marked by *no certain* external signs, up to the moment when the glands become tumid. The child may be fair or dark, pallid or ruddy, well fed, clad and lodged, or all these may be the worst possible; he may be the child of wealth or of poverty; he may live in town or country; he may be the child of old or young, of healthy or sickly parents; he may be born and live within the tropics, or in the arctic circle; under all these circumstances, as we shall see hereafter, scrofula may be developed." (p. 37.)

*The scrofulous deposit: its physical characters.* This is the subject of the third chapter. At its commencement we have at last a real definition of scrofula. "We," observes Mr. Phillips (meaning *I*, for only Kings and the Vehmgericht of the critics claim the dignity of the WE), "define scrofula to be a disease manifested by a peculiar deposit in the subcutaneous lymphatic glands." The qualities of this peculiar deposit are thus described by Professor Albers, Mr. Dalrymple, and Mr. Gulliver: the two latter made their observations by the special request of the author.

First, M. Albers:

"The tubercle presents, under the microscope, separate minute tubes, which under a linear enlargement of five hundred and fifty times, prove to be cells. This is not the case with scrofulous matter, which is granular." (p. 40.)

Secondly, Mr. Dalrymple:

"The whole material is composed of disintegrated tissue, granular molecules, irregular exudation-corpuscles, and in which the nucleolus is seldom to be recognized, and a considerable quantity of oil-globules, which may be abstracted by boiling in ether, and recovered by evaporation on a plate of glass.

"In acute or chronic inflammation of the glands, in otherwise healthy subjects, in whom no particular morbid disposition exists, the exudation-corpuscles, by what appears to be a law of vitality, proceed to the development of a cyst around the nucleus, or cytoblast; and this nucleus even splits into two or more, and hence a pus-globule is formed. At this point, however, the process stops, and the pus-globule subsequently disintegrates, and is resolved into granular and fluid matter. During the development of the cell and fissure of the nucleus, a pus-globule may be said to be an organic and vitalized body, deriving its means of increase from the blastema around.

"The exudation-corpuscle, however, is capable of a much higher degree of organization, and, under favorable circumstances, the cell-germ produces its cell, the cell elongates, and either fibre or filament is produced, as in the healing of a wound.

"In this scrofulous matter it appears that the exudation-corpuscles do not possess even that feeble vital power, which induces the further change into pus, and therefore it passes from the nucleolated cytoblast into an irregular granular body (disintegrated), the elements of which, by some further chemico-vital process, resolve partially into oil, or fat-globules." (p. 40.)

Thirdly, Mr. Gulliver—his observations upon tubercular and scrofulous matter have been very extensive:

"In the human subject, it appears to me that crude tubercular matter, from whatever organ obtained, differs as little in its microscopical as in its general and chemical characters. When examined by the aid of the microscope, crude tubercular matter can scarcely be said to present any regular structure, as it is merely made up of minutely granular matter, oily spherules, some shapeless albuminous flakes or shreds, and a few irregular corpuscles; the latter are probably nothing but effete, or shrunken primary cells." (p. 41.)

The chemical characters of scrofulous matters are not very clearly made out. It is certain, however, that a large proportion of albumen enters into their composition, and that they may vary considerably in the proportion of chemical elements.

*State of the organ in which scrofulous matter is about to be deposited.* It is well known that there is considerable hypertrophy of tissue and increased vascular action in the glandular structures previously to the deposit of the scrofulous matter. Mr. Phillips raises the question, whether this morbid state is dependent upon the blood circulating through the structures, or whether it is of local origin. Mr. Dalrymple thinks the glands are in an inflammatory condition. He thus describes the microscopic appearance of a scrofulous cervical gland.

"This enlarged gland appears to consist of a general parenchyma in a state of chronic inflammation, surrounding irregular masses of yellowish white matter more immediately the subject of examination. In direct proximity to the edges of this white material, the blood-vessels are seen to be more enlarged and congested than elsewhere, and in some parts, the capillaries are occluded with coagulated blood. The parenchyma, which at first sight appears healthy, is, on examination with high powers, found to be infiltrated with exudation-corpuscles, resembling lymph-globules. The natural texture of the gland consists of its proper corpuscles, filamentous tissue, blood-vessels, lymphatics, and nerves. In this morbid specimen, everywhere is the filamentous tissue infiltrated, and its fibres separated by innumerable exudation-corpuscles, and the proper corpuscles of the gland are similarly surrounded and imbedded. As the parenchyma is nearer to the white matter, so proportionally do the proper corpuscles of the gland become more indistinct, the filamentous tissue more obscure, the blood-vessels irregularly dilated and filled with red globules, and they at last disappear insensibly. The exudation-corpuscles are more numerous, but irregular in size and shape, and interspersed with minutely granular matter." (pp. 45-6.)

Mr. Phillips agrees with Mr. Dalrymple, and probably with the great majority of pathologists in the opinion that the glands are inflamed.

"This congested, inflamed, or hypertrophied condition of the subcutaneous glands is of very frequent occurrence. It can be detected in one fifth to one sixth of the juvenile population of this country. If we examine the necks of delicate children, we very commonly observe that the lymphatic glands of this region are enlarged. In the state of health, the eye and the finger fail to detect them at all, and when they are thus cognizable, the proof is complete that they have undergone the change of structure to which I have referred. A tumour of considerable size may result from the aggregation of a cluster, or a chain of such glands; that tumour may completely subside, so as to leave no trace of its existence behind it, and this is the result in probably nine cases out of ten where such swellings have been observed. This is proved by the fact that they are enlarged so as to be felt in more than 20 per cent. of the juvenile population in England and Wales, and that they are not found to proceed to suppuration in more than 2 per cent. of that population; and I regard this fact as a satisfactory proof of two circumstances—the hypertrophied or inflamed condition, on the one hand, and its entire subsidence on the other. And that subsidence I assume to be a proof that no scrofulous matter was deposited, because I have no reason to think that when scrofulous matter is deposited in any structure, it can be removed by any process of absorption; therefore, when the deposit has once taken place, the swelling it occasions must remain until the matter is ejected, because complete recession does not take place while the deposit is present in the part." (p. 46.)

It is generally in the largest and reddest gland, that the scrofulous deposit is found, but this is not invariably the case.

*Whence is the scrofulous matter derived?* Mr. Phillips investigates the theory of infarction of the glands by inspissated lymph, or chyle (in the case of the mesenteric glands). He sets it aside, however, for the more modern theory that morbid products are derived directly from the blood, and raises these questions: "Has the blood previously undergone a change in its elements, the part in which the deposit is made remaining unchanged? Or, does the blood remain in a healthy condition, the part in which the deposit is made exercising a specific influence upon the blood, and determining the character of that deposit? Or, are both removed from a healthy condition before the product is deposited?"

In answering the first question in the affirmative, Mr. Phillips displays, as indeed he does everywhere, great research both literary and experimental. The two following sentences contain the final results of his own investigations:

"I have examined the blood in sixty-seven instances of scrofula, and although I have almost always observed a considerable deviation from the condition of healthy blood, the changes have not presented sufficient uniformity to induce me to regard any particular condition as specially characteristic of scrofula; the changes are such as seem to belong to a tolerably extensive group of affections, all, it is true, being connected with disordered nutrition and debility . . . . . . I am not, therefore, in a condition to point out any particular state of the blood which is certainly characteristic of scrofula; but it is certain that if we examine the blood when the marks of scrofula are evident, its altered condition is also evident; but I know no particular condition of the blood which clearly marks the existence of scrofula. However, the occurrence of similar changes, at the same time, in different regions of the body, seems to point to some other than a local agent, as producing the change in the organ in which the deposit is made, and that agent the blood." (pp. 57-8.)

The "summing up" is given more decisively than is usual with Mr. Phillips, for, like many other philosophical inquirers, he has ofttimes kept us oscillating between two conflicting opinions, and left us at last in possession of neither.

"So much may, I think, be fairly assumed, that the blood is changed before the deposit is made, that the accumulation of certain morbid materials in the blood constitutes what is known as the scrofulous diathesis or constitution, and that their deposition in the subcutaneous lymphatic glands constitutes what we know as scrofula." (p. 89.)

*Are pulmonary tubercles, or phthisis, and scrofula in their nature identical?* This is the question Mr. Phillips has placed at the head of his sixth chapter. He answers it in the negative. In doing this, we are quite certain that he virtually opposes the opinion usually current amongst practitioners; but it is due to Mr. Phillips to say, that he has entered fully into the subject, that he has diligently sought for precise and numerical evidence, and that if he fail to convince his readers (as he has certainly failed to convince us) it is not for want of industry and research. We will let Mr. Phillips speak as much for himself as possible. His plan of inquiry is thus laid down:

"Our attention will first be directed to the characters of the product deposite in phthisis and scrofula, and the condition of the part in which the deposit is made; we will then proceed to ascertain whether the circumstances under which the deposit is made are identical; whether it occurs at the same period of life, and whether it fall with equal severity on both sexes; whether, where one of these affections is largely prevalent, the other keeps pace with it; and whether those who die of phthisis bear in their bodies the marks of scrofula; because if in most of these respects a kind of antagonism between these affections can be demonstrated, a conviction must then be induced, that how strong soever may be the resemblance in the general characters of those diseases, the points of difference are certainly not unimportant. And even if we should come to the conclusion, that in their nature these affections are identical, it might still be imprudent to lose sight of scrofula, as a term well known, to indicate a particular form of tubercular disease.

"First, we will consider the physical and chemical characters of the two products; and we will take tubercular matter, such as is found in the lungs, on the one hand, and scrofulous matter, such as is found in the subcutaneous glands, on the other, for the purpose of the comparison. I do this because we are considering whether phthisis, which I prefer to regard as a tubercular disease of the lung itself, and scrofula, which I define to be a disease of subcutaneous lymphatic glands, be in their nature identical. I exclude, therefore, from the present inquiry, disease of the mesenteric, the bronchial, and other glands existing in the thoracic or abdominal cavities, because I wish to consider phthisis and scrofula under their most decided characters." (pp. 61-2.)

We must really put in an initial *caveat* here. We can fairly require that words and terms be used in a precise and definite meaning. "I define scrofula to be a disease of subcutaneous lymphatic glands;" such is the precise meaning of the term scrofula as used in this chapter by Mr. Phillips. But is that the meaning in which it is used by pathologists and practitioners, or even by himself? We may most aptly quote Richard Wiseman, and appeal to the practitioners of this nation, whether physicians or chirurgeons, desiring them to say, whether there be not many tumours and ulcers commonly judged to be the evil, which are contained in no cystis at all, neither do they concrete into a glandule. We may also appeal to Mr. Phillips himself with the most perfect certainty of agreement, or else why has he placed "scrofulous bones or joints" amongst "the marks of scrofula"? More than two thirds of the cases of scrofulous disease of joints we have examined presented no enlargement of the subcutaneous lymphatic glands, so that if we adopt Mr. Phillips's definition, these cases so generally understood as scrofula ought not to be considered scrofulous.

We do not make these observations for the purpose of mere cavilling; they are necessary to the proper estimate of Mr. Phillips's statements and facts. In reality he has had it in intention to discuss the question generally on the wider basis, and we shall consider his facts accordingly; but in discussing the details, he first takes the more narrow definition and then the wider. For example, the pathological anatomy of scrofula as compared with that of tubercular phthisis, is limited to the morbid state of the subcutaneous cervical glands and the morbid product deposited in their tissue; there is no reference made to the pathological anatomy of other glands ordinarily decided to be the recipients of scrofulous deposit, as the bronchial, Peyerian, or mesenteric, nor of the inguinal and other subcutaneous glands. Neither have we any notice of the anatomy of scrofulous joints, when the scrofulous product is compared with tubercular deposit in the lungs. But in discussing the general pathology of the two diseases, Mr. Phillips adopts the wider definition of scrofula—that wider definition, indeed, on which the public and hospital statistics of the disease are founded.

It is but justice to Mr. Phillips, however, to state that he is himself aware of some discrepancy in his views in this respect, since he observes that "in its narrowest shape, the question is this, are tubercular disease of the lung and scrofulous disease of the subcutaneous glands identical in all other respects than in the seat of the deposit?" Yet even this is indefinite, and cannot be allowed to be an accurate exposition of the question. We must distinguish between the morbid change and the resulting symptoms; unless such distinction be rigidly made confusion is sure to result. The general symptoms of scrofula, taken rigidly in the meaning given to the term by Mr. Phillips, can only be those which result from softening and suppuration of the subcutaneous cervical glands in which there is a deposit of scrofulous matter; the general symptoms of tubercular phthisis can only be those of suppuration of the lungs in which there is a deposit of tubercular matter. In the latter case we know what these symptoms are; in the former we do not know, neither has Mr. Phillips informed us. The question that heads this sixth chapter is a significant example of this inaccuracy; "are pulmonary tubercle or phthisis and scrofula in their nature identical?" We understand from the text, as well as from the context, that the term pulmonary tubercle and phthisis are used convertibly; but it is known quite well that pulmonary tubercle may exist without the series of phenomena generalized under the term phthisis pulmonalis; phthisis may be latent, just as the scrofulous deposit may be present in the subcutaneous gland, unaccompanied by marasmus and hectic fever, or, in short, by any other appreciable pathological change in the organism. In following Mr. Phillips through his argument we shall endeavour to act upon the distinctions we have thought it necessary to make, and thus more clearly elucidate the subject itself.

*Is there any difference between the scrofulous deposit in the subcutaneous cervical glands and the tubercular deposit in the lungs?* No very remarkable difference is perceptible by the naked eye, except such as may be dependent upon the difference of structure of the two organs. There is no essential difference in their chemical elements. Sandras and Albers believed that they detected a microscopic difference between the two products, but Mr. Gulliver controverts their statements. He says:

> "In the human subject, it appears to me, that crude tubercular matter, from whatever organ obtained, differs as little in its microscopic as in its general and chemical characters. The drawing [a plate is appended to the work] shows how nearly the microscopic elements composing crude tubercle of the lungs and of the lymphatic glands agree. All the objects are magnified about six hundred and eighty times, linear admeasurement. In the upper part, *a*, is depicted tubercle from the lung of a man, aged twenty-four, who died of the disease; in the lower part, *b*, tubercle from a subcutaneous gland of the neck of a boy, aged nine, who died of scrofula affecting the superficial glands, membranes of the brain, and the spleen; his lungs contained only a few small tubercles." (p. 64.)

The result of Mr. Phillips's own judgment on the facts and statements investigated by him is, that in so far as concerns the physical and chemical qualities of scrofulous and tubercular matter, we can, at present, draw no distinct line of difference between those products.

*Is there any difference in the part in which the deposit is made?* Mr. Phillips says there is. The scrofulous deposit is preceded by inflammatory action; tubercular deposit is not preceded by inflammatory action. In this respect he insists there is a marked difference between the two, and that it is an important difference. The proposition is open to considerable doubt, but if it be granted, it can only be granted as a specific difference dependent upon the difference in structure between the lymphatic glands and the lungs; the deposit itself and the diathesis in which that morbid product originates may be identical. Mr. Phillips decides in the negative as to any difference as to vascularity of the morbid products: neither can be injected.

*Difference as to the period when scrofula and phthisis destroy life.* Here Mr. Phillips uses the term scrofula in the wider sense, and adduces the mortality tables to prove "that the generally received opinion is correct, that the ravages of scrofula, where it destroys life, are most severely felt before, those of consumption after, the period of puberty." In the metropolis during the year 1840-1, 21 per cent. of the persons who died of consumption were under fifteen years of age, while 60 per cent. of the deaths from scrofula were under that age. A comparison of other districts in England exhibits similar results, and induces Mr. Phillips to state:

"The proof, then, seems to me ample, that the period of life when the deaths from phthisis are most numerous, is not that when the ravages of scrofula are most keenly felt; and this constitutes another distinction between these diseases. I know that it is said that the registered deaths from scrofula are no proof of the extent to which it prevails, but such reasoning would also apply to phthisis. Complete evidence they may not furnish; but as approximative evidence, they are unexceptionable. If the deaths from scrofula in a given district be 100, and in another district of similar population 50, I conceive it would be abundant proof of the greater prevalence of the disease in the former district than in the latter; though it might not show how far enlarged glands could be detected in either case." (p. 71.)

We have considerable doubts as to the scientific value of the facts on which Mr. Phillips founds his numerical estimate. By "deaths from scrofula," he cannot mean deaths from scrofulous disease of the subcutaneous lymphatic glands, for it is not probable any person has ever died from that disease; nor can he mean deaths from diseases in which enlargement of the gland is a pathognomonic sign. We know positively that the term scrofula is used by medical practitioners in a much wider sense than this; and as defined in the 'Statistical Nosology,' published by the Registrar-General, it includes chronic abscesses of all kinds, as psoas abscess, abscesses of the skin not glandular, and suppurating joints of every kind. If we adopt these latter affections into the definition of scrofula, then scrofula is "a disease of the constitution," and Mr. Phillips has stated no solid grounds in proof of his proposition, that a deposit of scrofulous product in the cervical glands is the only pathognomonic sign of this affection. The old maxim of *ubi irritatio ibi fluxus* is substantially true, and we can have no difficulty in conceiving that the scrofulous matter already circulating with the blood may be deposited in other glandular and parenchymatous structures than those of the superficial cervical glands. Indeed, seeing that the fluid from whence the product is derived pervades the whole system, it seems altogether unreasonable to limit its deposit to such comparatively unimportant structures as the subcutaneous glands of the neck. On the contrary, we may fairly assume from a consideration of all the laws regulating the deposits of nutritive, excretive, or morbid matters from the blood, that such exclusive deposit does not take place.*

Assuming, then, that the true nature of scrofula is a dyscrasy, or morbid condition of the blood, and that the scrofulous product is deposited from the latter, we shall have a family of scrofulous diseases differing in their pathological anatomy and their semeiology, according to the sex, the age, and the organ or structure in which the scrofulous product is deposited. Considering the question in this way, we are decidedly of opinion that the mortality tables cannot afford data for a scientific inquiry into the pathology of the two forms of disease in question. We shall, however, subsequently show that the deductions they do afford when properly estimated are altogether opposed to those of Mr. Phillips; we think they prove the identity of the two diseases, rather than their distinctness. We may here state, that the differences as to the sex of the victims of phthisis and scrofula is also in favour of our views. The identity of the two diseases by no means demands that they should affect both sexes with equal severity. The sexes are under different predisposing circumstances. In early childhood and youth, the greater activity of the boy renders the joints more liable to those injuries which so frequently induce scrofulous disease in them. On the other hand, in the pubescent female, the energy of development is expended rather in the pelvis than the thorax; being the converse of what happens in the male, and hence we should anticipate more cases of pulmonary deposit in the former sex than in the latter. Now this is precisely the result of Mr. Phillips's statistical inquiries. By data obtained from the Registrar-General's Reports for 1839-42, he shows that there was a preponderance of female mortality from phthisis in England and Wales of 15 per cent., but that the mortality of males from scrofula (that is, *scrofulous diseases*) exceeded that of females by about 24 per cent. In Ireland, the deaths of females from phthisis exceeded those of males by 13 per cent., but the deaths from scrofula (*scrofulous diseases*) were fewer by 35 per cent.

The difference in the relative prevalence of the two diseases is illustrated in a very striking manner by Mr. Phillips. But here again we must caution our readers as to the meaning attached to the terms "scrofula" and "phthisis" or "consumption" in the statistical nosology; the former we again assert comprises a *class* of diseases and the latter includes other forms of chronic pulmonary disease than tubercular phthisis. With this *caveat* we subjoin Mr. Phillips's facts in his own concise way.

* M. Louis expresses an opinion of this kind so forcibly, respecting the deposit of tubercular matter, that we are induced to quote it: "Whenever these products presented themselves, I have never observed them in any viscus without their being likewise present in the lungs. I speak, be it remembered, of observations made on individuals who had passed their fifteenth year. Whence the apparent conclusion that their existence in the lungs forms a necessary condition for their development in other parts. There is another fact which appears further to demonstrate the reality of this connexion between the lungs and other organs, in respect of tuberculous development, or at least the agency of some one single cause acting at the same time on a great number of parts of the body. It is that, with the exception of a solitary case, I have always found the tuberculous deposit in a state of greater advancement in the lungs than elsewhere, and that, when tubercles existed in various other parts of the system besides the lungs, they had in all those others attained the same amount of development. Now, it would be difficult to conceive this uniformity in parts the most remote, and the most different in structure from each other, without admitting the action of a single cause, acting simultaneously on a great number of parts, and quite independently of the accidental influences which we may in some cases be induced to recognize." (Researches on Phthisis. Sydenham Society's edition, p. 153.)

"The Registrar-General's Tables for England and Wales, if we take a period of four years, show that the deaths from consumption amount annually, on an average, in round numbers, to 59,500; those from scrofula to 1200; the proportion which the one bears to the other is as 1 to 50; and the proportion they severally bear to the gross population as 1 to 265, and 1 to 13,255 for England and Wales. If the causes of the two diseases be the same, that proportion should obtain in each district; let us see whether it does. In the north-west district, the deaths from consumption are 9976, those from scrofula are 116; the proportion which they bear to each other is as 1 to 86, and to the gross population as 1 to 206, and 1 to 17,782. Here, then, we see that, at the same time, the causes of consumption are in action with more, whilst those of scrofula exhibit 20 per cent. less, intensity than the average of England and Wales. If we take the town population selected by Mr. Farr, in page 198 of his fifth Annual Report, including four years, ending in 1842, we find a population of 3,759,186. The deaths from consumption are to the population as 1 to 235, while those from scrofula are as 1 to 20,000. If we take his counties, in the same page, we get a population of 3,446,501. The deaths from consumption amount to 1 to 286, those from scrofula to 1 per 10,000, or 100 per million. The deaths from consumption being 19 per cent. greater in the town than in the country districts, while those from scrofula are 100 per cent. less.

"The evidence to be obtained from the mortuary tables of our own country proves, therefore, that if the causes of the two affections are the same, the effects differ most widely; that where there is a large mortality from consumption, there is a small mortality from scrofula, and *vice versâ*." (pp. 72-3.)

The antagonism as to the prevalence of these diseases (or rather two forms of the same general disease) is further demonstrated by continental statistics and facts. In India consumption is rare; scrofula rife; so too in Russia. In the latter country, and also in Austria and Bavaria, those who bear about their necks ugly badges constituted by scrofulous ulceration are supposed to be exempt from consumption. This fact is remarkable, and Mr. Phillips is of opinion, that it shows that no necessary connexion is thought to exist between the two diseases; but may it not point out an intimate connexion? May not in fact it be, that the scrofulous diathesis once passed safely through will not readily occur again? Is it possible that one attack of scrofula may, like an attack of smallpox or scarlatina, afford a greater or less immunity from another? There is nothing at least outrageously improbable in the supposition.

It is remarkable that some facts stated by Mr. Phillips on this point are in favour of this view. In opposition to the objection that the registries do not show the amount of scrofula in a district because scrofula does not commonly destroy life, Mr. Phillips observes that if the sufferer from scrofula usually dies of phthisis there should be the marks of the former in the bodies of those who die of the latter. Now, we think this is by no means a requisite, so far at least as regards the traces of previous suppuration of the cervical glands, for we hold that (uncomplicated with any other form) to be the simplest and most harmless species of scrofulous disease. The facts, however, though collected to elucidate an opinion which we cannot well admit, are not the less valuable, and they show that a previous attack of scrofula in so far as it is indicated by marks, does not predispose to a second attack. Of 332 cases of phthisis examined by Mr. Phillips at the St. Marylebone Infirmary, only 7 presented scars resulting from scrofula!

In seven of the 332 cases just mentioned there was tumefaction of the cervical glands, but in only two instances did they contain scrofulous matter. On this point of the pathology of phthisis and scrofula Mr. Phillips quotes M. Louis, who, in 350 phthisical bodies carefully examined by him, discovered a tuberculous condition of the cervical glands in only 35. From these and similar facts, Mr. Phillips draws another argument against the identity of scrofula and phthisis. We cannot, however, admit its conclusiveness. In scrofulous diseases of the joints we have enlarged cervical glands in probably not more than 30 per cent., yet Mr. Phillips would not we think argue that no diseased joint can be pronounced scrofulous unless the cervical glands also are affected with the scrofulous deposit. If he do, the *whole* of his statistical returns are invalidated, and of no worth.

We should be better able to say why the lymphatic glands in cases of tubercular phthisis and scrofulous diseases of the joints are not usually the seat of scrofulous product, if we were better acquainted with the circumstances which determine its deposition. In the class of diseases alluded to, it is not improbable that the morbid condition of the organs implicated determines a vicarious action in them, which prevents deposition in adjoining structures. Such an action is seen in the metastasis of arthritic and rheumatic diseases, and may occur also in scrofula. Thus, according to Hasse, whose deductions are made from 245 examples of phthisis, the bronchial glands are tuberculous in only 26 per cent., while the mucous membrane of the larynx and trachea are the seat of *tuberculous* (not "scrofulous") deposit in only the same proportion as the cervical glands, namely, 5 per cent.! The *class* of tissues may also exercise an influence in determining the deposit.

It appears then that the tubercular deposit in the lungs and the scrofulous deposit in the subcutaneous glands do not differ either chemically or physically; that the difference in the semeiology of tubercular phthisis and scrofula is solely dependent on the difference in the class of organs attacked; and that the diversities as to age, sex, and character of the population attacked are caused by a difference in the *predisposing* or *exciting* causes; the *proximate* cause being the same in both, and consisting in the deposit of a similar morbid product from the blood.

*Prevalence of scrofula in Great Britain.* An opinion is very generally entertained that scrofula is pre-eminently an English disease. Mr. Phillips addressed himself to the task of determining how much of truth the opinion might contain. He had a large number of children examined in schools, union houses, factories, and elsewhere. Of 133,721 examined, in many districts 24½ per cent. presented the marks of scrofula (scrofulous disease) indicated in Mr. Phillips's form of return. But the gross number of cases in which the disease so marked was obvious to the eye amounted to not quite 3 per cent. In union houses the proportion was rather greater, in charity schools rather less. All things considered, Mr. Phillips thinks "that the proportion of children between the ages of five and sixteen who present marks of scrofula evident upon a simple inspection amount to as near as may be, but rather under 3½ per cent." The proportion of adults is much less. Of 1521 examined, only 23 had such marks. Of cases of diseased children treated at dispensaries and hospitals, Mr. Phillips estimates 4 per cent. to be cases of scrofula, and of the gross number of persons treated about $1\frac{1}{4}$ per cent. are scrofula. Of 95,586 recruits examined 1 in 119 were rejected for marks of scrofula. We subjoin Mr. Phillips's summary on this point; but we observe, by way of caution, that the number of persons exhibiting external and visible signs of scrofula is not an index of the prevalence of *scrofulous diseases,* but of the prevalence of those forms only which affect external organs. We may add too that all the children *under* five years of age are taken no note of.

"It is thus seen, that though derived from so many and such different sources, there is a striking concurrence in the results of the evidence I have collected, and that agreement constitutes a strong reason for believing that my data do very nearly represent the actual prevalence of the disease. We see that the returns of cases of scrofula, found among our ordinary population, are singularly confirmed, not only by the returns of hospitals and dispensaries, but also by the examination of recruits and convicts; and I think we are thus justified in regarding as near the truth our estimate of the prevalence of scrofula, such as we have defined the disease. That is to say, that scars are apparent in about $1\frac{1}{2}$ per cent.; that the subcutaneous glands are enlarged, so as to be perceptible on simple inspection, in less than 3 per cent.; and that the glands may be detected by the finger in $24\frac{1}{2}$ per cent. of those of the children of the poor who are under sixteen, and in 8 per cent. of those above; or taking the whole population, in 10 per cent.; and that something less than 3 per cent. of the people are under treatment for the disease in its various forms." (p. 84.)

*Unequal prevalence of scrofula.* Mr. Phillips's extensive returns have enabled him to compare the state of the population in different portions of the British Isles as regards the prevalence of scrofula, and the result of his inquiries is, that it prevails very unequally. In some districts, 72 per cent. of the children of the poor presented marks of scrofula, while in others the proportion was so low as 11 per cent.

"If we take two agricultural districts, the Eastern, which comprises Essex, Suffolk, and Norfolk, and the South Western, which includes Wilts, Devon, Dorset, Somerset, and Cornwall, we find in the former that the deaths from scrofula are 11·8; and in the latter as 10 to every 100,000 population, being a difference of nearly 20 per cent. In the purely manufacturing districts of Lancashire and Cheshire, the proportion is only as 5·6; while in that of Yorkshire as 3·5 per 100,000; and in the mixed districts of Gloucester, Hereford, Shropshire, Worcester, Stafford, and Warwick, it is as 7·9 to 100,000.

"The Irish census returns, including as they do a period of ten years, show a mortality from scrofula of 1 in 25,252 of the population; but in the different provinces there is considerable variety. Thus in Connaught, it is 1 in 21,177; in Ulster, 1 in 31,399; in Munster, 1 in 23,363; in Leinster, 1 in 29,025; but to this mortality should be added much for marasmus. In the case of the Irish returns, the deaths from marasmus are very large, amounting to 6,865 annually; but as many of these may have been cases of scrofula, the absolute value of the returns, in so far as concerns consumption and scrofula, is much lessened.

"The infirmary and dispensary returns in Ireland show a proportion of scrofulous cases amounting to 82,746, of these 1290, or 1·5 per cent. are registered scrofula. The examination of children in Limerick gives a proportion of 5·4 per cent. having the ordinary marks of scrofula. And the total deaths comprised in Dr. Griffin's paper were 2918, those from scrofula 7.

"The recruiting returns, for the United Kingdom, show an average rejection of 8 per 1000 for marks of scrofula. In England the smallest number of rejections for scrofula occurred in London, where they amount to 5 per 1000. In Scotland, in the Edinburgh district, 16 per 1000; in Ireland, in the Dublin district, 13 per 1000." (p. 87.)

Mr. Phillips thinks these statements constitute a near approximation to the truth. The caution which preceded our last quotation applies also to this.

The prevalence of scrofula (as indicated by external marks) in other countries, as compared with England, presents some interesting points of comparison. In the Orphan Asylum at Lisbon (as we learn from the returns procured by Mr. Phillips) Dr. Rosas examined 800 children, of whom 279, or 35 per cent., bore the ordinary marks of scrofula. Of the boys the proportion was 50 per cent. of the whole number examined; whilst of the girls it was only 10 per cent.; a fact remarkably in accordance with the principle we laid down (p. 136) that the identity of two diseases by no means requires that they should affect the two sexes with equal severity. In the Orphan Asylum at Amsterdam, of 495 children, 209, or 42 per cent., bore the ordinary marks of scrofula. At the Orphan Asylum, Munich, the Report shows that scrofula, at one time, affected two thirds of the children, but under improved diet and air the disease had lessened in frequency. The Vienna returns show that the number of children boarded in the Imperial Royal Orphan Asylum was $\frac{308 \text{ boys}}{104 \text{ girls}}=412$; of these, during the year 1841, $\frac{18 \text{ boys}}{27 \text{ girls}}=45$, or 11 per cent., came under treatment for scrofula. The return made by the President of Police at Berlin, Von Puttkammer, of the condition of the children who were within the walls of the Frederick Orphan Asylum, shows that the number was $\frac{230 \text{ boys}}{123 \text{ girls}}=353$; of these, 125 boys and 50 girls had enlarged glands, 4 boys and four girls had scrofulous ulcers, 2 girls had scrofulous joints; making 185, or nearly 53 per cent. At St. Petersburgh, of 840 children examined, 343, or nearly 41 per cent., bore evident marks of scrofula. At the Imperial Foundling Hospital, at Moscow, the number of children examined was 15,515; of whom 1294, or only 9 per cent., are reported as presenting tumid glands, ulcers, and sinuses resulting from scrofulous swellings and diseased joints. This proportion, Mr. Phillips observes, is very small; and that trifling enlargements have been passed over is rendered probable by the fact that the number presenting scrofulous ulcers and sinuses, and diseased joints, is greater than the number which has been reported to possess tumid glands, the latter being 583, and the former 711. There is, therefore, little doubt but that in the whole of the cases the disease was evident to the eye, and, if so, the proportion was very large; and even if we take the 711 cases only in which we know the disease was apparent to the eye, the proportion is large—nearly five per cent.

The returns from America varied greatly, and are evidently fallacious.

"That from the Boston House of Industry includes $\frac{98 \text{ boys}}{48 \text{ girls}}=146$ children; of whom 106, or 70 per cent., are returned with the specific marks of scrofula. That from Philadelphia, carefully supervised by that able physician, Dr. S Jackson, shows a total of $\frac{2641 \text{ boys}}{1357 \text{ girls}}=2998$ school children examined; of whom $\frac{10 \text{ boys}}{3 \text{ girls}}=13$ are returned as scrofulous......Taking the population of New York, in 1840, at 345,000 in round numbers, and taking the deaths from scrofula as reported, at the average of 35 years, at 4278, we find that the annual deaths from scrofula bear to the total population a proportion of 1 to 1241. In Philadelphia the proportion is 1 in 453; while in London, in the same year, the proportion is, in round numbers, as 1 to 17,500." (p. 88.)

The prevalence of scrofula in Syria is less marked than in Great Britain. Dr. A. Jackson states that he thinks in India 80 per cent. of half-caste children are scrofulous, 50 per cent. of natives, 40 per cent. of English, and 10 per cent. of Mussulmen.

From the mortuary tables of Paris and Geneva, Mr. Phillips gathers that the mortality from scrofula is much greater in those cities than in our own. Taking a series of years, the deaths from scrofula in Paris amount to 1 in every 3221 of the population; in Geneva, to 1 in every 2790; in the year 1842, taking the population at 61,871, and the deaths from scrofula at 16, the proportion is 1 in 3867, while in London, taking four years ending in 1842, they amount to about 1 in 9,000.

We think that before full reliance can be placed upon these statistics, it ought to be shown that the nosology of the registrars in the two countries is similar. If tabes or marasmus of infants be entered as scrofula in Paris or Geneva, the statement will assume a very different aspect.

Taking the whole of France, the rejection of conscripts for marks of scrofula are 2 per cent., or 1754 in obtaining 86,000; our own proportion is 1 in 119. The rejections for scrofula in the Département du Nord are 46 per 1000; in the Eastern Pyrenees, 1 per 1000. Mr. Phillips infers that for the whole of France the marks of scrofula presented by recruits are twice as many as among our own recruiting population. This conclusion, if it means that scrofula is twice as prevalent in France as in England, is certainly erroneous. The strength of the French army is kept up by a forced conscription and not by recruiting, and, consequently, the circumstances under which the two classes of military candidates are presented for approval are totally dissimilar.

Mr. Phillips finally inquires, "Is it not, then, abundantly proved that the notion that scrofula is eminently an English disease is incorrect? and am I not warranted in stating that there is no country, so far, at least, as our information extends, in which the people are more free from the disease than in England and Wales?" We scarcely can grant it "abundantly proved," or even proved at all. There is a fair presumption, however, that the fact is so.

Mr. Phillips devotes a chapter to the question whether scrofula be now more prevalent in England than formerly. He enters into some statistical details, which must have cost him much trouble in collecting, and some of which are quite original, especially those respecting the numbers "touched" by royalty. Mr. Phillips is of opinion that scrofula prevails much less extensively now than in the 17th and 18th centuries.

*Causes of scrofula.* We now come to a much discussed question,—What are the causes of scrofula? They have been sought for in hereditary transmission, contagion, defective alimentation, imperfect aeration, deficient clothing.

*The hereditary cause.* The doctrine that scrofula (or scrofulous diseases) may be transmitted hereditarily is founded upon both theory and experience. No one can pay ordinary attention to the physiology of propagation of animals without noticing that the most minute characteristics of parents are transmitted to their offspring. Hence it is fairly argued that if any of the structures are so constituted in the parents, that scrofulous products shall be formed on a sufficient exciting cause occurring, such a constitution may be certainly expected in the offspring, as certainly as the characteristic form or individual features.

Observation has shown, too, that it is not always the features of the parent, but often those of the grand-parent, that are reproduced in the offspring. Such facts are well known to the breeders of domestic animals, who term the process "breeding back." Hence it has been inferred that the morbid predispositions of the grand-parent, and not those of the parent, may be reproduced in the offspring. The inference that a morbid predisposition of parent, or grand-parent, may be transmitted to offspring, is founded upon the axiom that the whole is equal to the sum of all its parts; that as every line and fibre that make up the parent are represented in the offspring, so the qualities of every tissue will be represented.

It has been usually granted that those characteristics which mark the species, and are natural to it, are transmitted to the offspring; but it has been doubted whether acquired characteristics, or acquired predispositions, can be transmitted. But of this fact there can be no doubt: the pups of well-bred pointers will point like their parents, before they have received a lesson. Examples of a similar kind might be multiplied. The fact of hereditary transmission of acquired instincts, or predispositions, is certain; it only remains to determine the limits within which the law operates, and which are as yet indeterminate.

Holding it, then, as established that a predisposition in the tissues to disease may be transmitted, it remains to determine whether a disease with which one or other of the parents was affected at the time of conception of the offspring, or the mother during the period of utero-gestation, can be transmitted. This is certain; but the limits within which this law operates are also, as yet, indeterminate. We know that smallpox and syphilis may be transmitted under these circumstances; and it is not improbable that examples of transmission of other diseases may be detected on more minute observation, and particularly of those diseases which are dependent on an animal poison. But we think that it is not necessary to the production of scrofula, or of any other constitutional disease in the offspring, that one or both the parents should have been suffering from the disease at the time of conception of their progeny. There are grounds for believing that a previous impregnation at a time when one or other parent was actually diseased, will exercise an influence on subsequent conceptions. A seven-eighths Arabian mare, belonging to the Earl of Morton, had her first foal by a quagga; subsequently she had three other foals by a black Arabian. Now, the first two foals of these three by the Arabian had a striking resemblance to the quagga in the markings of their coat and in the form of their mane. Similar facts might be adduced.

On a careful perusal of Mr. Phillips's observations on this point, as respects scrofula, we feel inclined to believe that he has carried his numerical scepticism too far. Probably too great stress has been laid upon the hereditary transmission of diseases, or the predisposition thereto, but the fact of such transmission is based on a much higher order of proof than fallacious statistics. Even in reference to insanity, observes Mr. Phillips, "the case of all others in which the truth of the hereditary influence might be most easily tested, and in which the conviction of its existence is, perhaps, the strongest, I know no conclusive evidence." But has Mr. Phillips carefully looked for it? It must be observed that in an investigation of this kind positive facts must be admitted to have a most preponderating weight. "Of 191 patients admitted into Bethlehem Hospital, in 1844, an hereditary cause could only be discovered in 26 cases." Such is an example of the facts on which Mr. Phillips grounds his scepticism. At best it is only negative. But is not such a small proportion (so decidedly at variance with all experience) a warranty rather for doubting the acumen of the observer, than the known and long-cherished dogma? Of 415 insane persons admitted into the Retreat, near York, since its foundation, and connected with the Society of Friends, the disease was connected with an hereditary predisposition in 142 cases, or in the proportion of 1 in 3. And of the remaining 273 cases there were, doubtless, some in whom the hereditary predisposition existed, although not made out. Now, unless Mr. Phillips is prepared to assert that insanity is a natural and spontaneous disease in one of every three families of the Society of Friends, such a connexion between it and the hereditary predisposition must be something more than a mere accident; there must be a causal relation. With regard to scrofula, our author has instituted some important and extensive inquiries; an account of which we subjoin:

"The means which I have taken to acquire accurate data as to the extent to which hereditary causes operate, in the propagation of scrofula, are the following: I examined myself, and procured to be examined by others, in the metropolitan, the factory, and in rural districts, upwards of 2000 families, each consisting of from three to five children, and living, as nearly as may be, under similar circumstances. In one portion of the cases, both parents were apparently free from scrofulous taint; in another portion there was reason to think that both parents were tainted; in another, that the father was tainted; and in another, the mother. The number of families examined was 2023, the number of children was 7587; and the number bearing such marks of scrofula as I have already indicated was 1738, or nearly 23 per cent. In 506 instances, derived from many localities, and under the most varied circumstances, both parents were apparently untainted, and their offspring amounted to 2021. Of these, 421, or something less than 21 per cent., presented marks of scrofula. In 276 instances, there was reason to think that both parents laboured under scrofulous taint; their offspring amounted to 1092 children; of these, 271, or nearly 25 per cent., bore the ordinary marks of scrofula. In 589 instances, the father carried about him marks of having suffered from scrofula, whilst the mother was free from them; their children amounted to 2107, those having marks of scrofula to 483, or nearly 23 per cent. In 652 instances, the mother bore upon her person the marks of scrofula, whilst the father did not; their children amounted to 2367, and of these, 563, or nearly 24 per cent., presented marks of scrofula.

"In glancing over these results, it must be kept in mind that the offspring of the tainted, on the one hand, and of the untainted, on the other, are not intended to represent their relative fecundity, for means were taken to collect only such families as were represented by not less than three, nor more than five children. .......... I do not pretend to regard these results as an accurate representation of the influence of scrofula when existing in the parent to reproduce itself in the child. I would even admit that, as the cases were seen with many eyes, the data may be more defective than if they had been the result of one person's examination; but however defective they may be, they are the only approach I know of to a reasonable amount of evidence, to enable us to judge how far it is probable that scrofula in the parent will reproduce itself in the child. And from that evidence it would seem that in children subjected after birth to similar circumstances, the hereditary influence does not appear to be exerted beyond 4 per cent. This result is in opposition to two parties—one maintaining that the disease is always hereditary, and never acquired; the other, that no diseases are hereditary, but that they are always the result of circumstances which come into action after birth." (pp. 118-20.)

We fully participate in the sentiments expressed by Mr. Phillips as to the imperfection of the data here given. The whole question is so complicated that its solution by such simple numerical results is not to be expected.

*The question of predisposition.* We judge from the context that Mr. Phillips questions the actuality of an hereditary predisposition to scrofula. We cannot say that we participate in his doubts, although he is quite correct in stating that it is difficult to test numerically. It may even be doubted if the proofs can ever be derived so clearly from statistical inquiries as from ratiocination on observed facts. We see the children of highly scrofulous parents, who are not simply of a weakly constitution, but who are truly scrofulous, and who become so, although placed under the most favorable circumstances for prophylaxis and the preservation of health. Such families come occasionally under the notice of most practitioners, and furnish extreme instances of the law of hereditary predisposition. This predisposition to scrofulous deposit has its analogue in the hereditary predisposition to arthritic deposit. As in the highly scrofulous families, so with highly gouty families, we find the hereditary predisposition develops the disease, in spite of the most careful prophylaxis. This is a matter of fact. We have ourselves carefully noted it. But in all these examples of predisposition to a particular family of diseases, whether they be the neurotic, the arthritic, or the strumous, the predisposition varies in every degree from the two extremes. The scrofulous predisposition may be so slight as to be developed into scrofula when the causes which excite it into activity are so powerful that they would develop like diseases in an untainted constitution; or the proclivity to scrofulous deposit may differ in different organs in the same individual, so that, although in childhood the lymphatic glands may escape all disease, at puberty the lungs will be inevitably involved.

*Will any other disease in the parent develop scrofula?* There can be no doubt that a delicate constitution favours the development of scrofula, whether that be derived from parents, or be a deteriorated constitution. Long continued indigestion in a parent seems to favour the development of scrofula in a child, as well as the other causes enumerated by Sir James Clark, whose opinion Mr. Phillips controverts. In doing this, he observes that the marks of scrofula do not commonly appear during the first two years of life. This is scarcely, we think, correct, even if the marks of scrofula be allowed to be none other than those which Mr. Phillips has laid down, but decidedly incorrect, if we consider disease of the mesenteric glands a mark of scrofula. We are more in accordance, however, with our author in his views as to the independence of scrofula and syphilis; indeed we fully concur with him. It appears to us that much error has arisen with regard to the relationship between the two diseases, from the fact, that persons of scrofulous constitutions suffer severely from secondary syphilis, especially from syphilitic affections of the throat and bones. This probably results from the circumstance that the same class of tissues is the seat of both diseases.

Mr. Phillips thinks the children of old men are more likely to suffer from scrofula, but principally because they are weakly, and not because there is any power in the debility of age to transmit the scrofulous diathesis. He also thinks that too much injurious influence has been attributed to intermarriages; at least they do not tend among healthy persons to the production of scrofula.

*Can lactation by a scrofulous nurse develop scrofula in the child?* or can it be communicated by contagion? or by inoculation with pus taken from a scrofulous person? Mr. Phillips answers all these questions in the negative. It does not seem probable *a priori* that vaccination from a scrofulous child will communicate scrofula to a healthy one, but it is far from improbable; indeed we should feel inclined to think it certain that vaccinia, or variola, may exercise such a powerful influence on the mucous tissues as to develop the disease, if it do not already exist; or (paradoxical as it may appear) to remove it, if it do. We know very well that many remedies display this antagonism of action, and we need not deny the same mode of action to morbid poisons. It need not, however, be granted that variola and pulmonary tubercle mutually repel each other (as Rilliet and Barthez think), because there has been a larger proportion of phthisical cases since vaccination was practised; it is a more reasonable inference that by an improved hygiene, scrofulous children have been enabled to survive until puberty, then to die of phthisis.

*General causes of scrofula.* These are specially considered under the heads of food, air, climate, humidity, temperature, occupation, &c. In discussing the question of defective nutrition as a cause of scrofula, Mr. Phillips refers extensively to the statistics of foundling hospitals, especially those of the Continent, to demonstrate that the nature of the food administered to infants has a most important influence on their health; and that the bringing up a child "by hand," as it is termed, conduces to a high infantile mortality. If the food be not suitable to the infant, or if it be taken in an unnatural manner, it acts injuriously upon nutrition. It appears, indeed, that a larger proportion of scrofulous disease is found among children who have been brought up by hand than among those who are suckled by a mother, or even a foster-mother; and that, therefore, the system of bringing up children by hand deranges nutrition, and adapts the constitution for the deposit of scrofulous matter in the child.

In order to test the influence of food on the development or prevention of scrofula, Mr. Phillips collected extensive materials for a comparison between children in union workhouses, or in certain charitable institutions, and a similar class not so well fed at their own homes. The author's summary of his results is as follows:

"To ascertain how far scrofula prevails in union workhouses, 9342 children, the inmates of 63 houses, in different districts were examined; and of these 2139, or nearly 23 per cent., presented marks of scrofula.

"In a large town I examined 784 children, in endowed and other schools, who, for the most part, had been well cared for before admission, and judiciously managed afterwards; and the 'marks of scrofula' could be detected in 127 instances, or 16 per cent.;—500 children in national schools, brought up at home, of whom the most part were not well fed and lodged, and of these 164, or 32 per cent., bore similar marks of scrofula. And 500 children examined in the workhouses yielded 126, or 25 per cent., bearing marks of scrofula.

If we compare the children in the St. Marylebone workhouse with the children of the national school in High-street, St. Marylebone, there will be no question of the physical superiority of the former; for while the children in the workhouse are affected with scrofulous swellings to the extent of 26½ per cent. only, those of the national school are affected in the proportion of 32 per cent.

In the same districts with those of the 63 union-houses, 22,704 children living at their own homes, or in institutions other than poorhouses, have been examined; of these 8,353, or 37½ per cent., had marks of scrofula.

The dietaries of union workhouses, as contained in the Poor-law circular, having been compared with the food of the agricultural labourer, and the prevalence of scrofula in each condition having been shown, we arrive at this result, namely, that the workhouse child is better fed, and less subject to scrofula than the child reared in the cottage of the peasant; and when it is considered that many pauper children are received into the workhouse when suffering and destitution have probably developed disease, and have almost certainly produced debility, the comparison is even less favorable for the child of the independent labourer, than the result which is shown by the numbers actually examined. Now, with the exception of food and clothing, the workhouse child enjoys no advantage of which the child reared in the cottage is deprived. The rooms he inhabits may be larger and better ventilated, but a greater number of persons is collected in these rooms, and the breathing space for each person may even be less than in the narrow limits of the cottage." (pp. 171-73.)

The result of Mr. Phillips's inquiries in Ireland, on this point, and amongst the schools both on the Continent and in the United States, are detailed also at length.

*An impure atmosphere as a cause of scrofula.* Mr. Phillips thinks it due to his subject to enter upon a discussion of the question how far the overcrowding of the population in large towns is detrimental to health. We do not think our author handles his statistics cleverly in this section. For example, he argues that if density of population be assigned as an exclusive cause of the excessive mortality of towns, it should be found to act uniformly, and the mortality should be in a direct ratio to the density. But it is not so. The metropolis has a denser population than Bristol or Leeds, yet the mortality is less. Now a little consideration might, we think, have led Mr. Phillips to the conviction that the term density of population is extremely vague. It is well known to practical inquirers into the sanitary condition of the people, that the indefinite use of the term has led to the grossest mistakes. A population may be crowded into narrow courts, and be as densely packed as possible, yet because of the surrounding superficies being surrounded by factories, warehouses, streets, squares, &c., the number of people, *per square mile,* may be inconsiderable. The plain truth is, that the attempt to ascertain the effects of overcrowding, or of uncleanness, or of poverty, from the bare statistics of the public registries, is altogether vain. The more experience we have of statistical deductions, the more convinced we are of the multitudinous fallacies to which they give rise, except when they are thoroughly sifted, and studied in all their relations, by persons conversant with the particular details from which the data are taken. That pure air is necessary to health we know certainly; that the air of an apartment occupied night and day by a family, situate in a confined court, surrounded by high buildings, and ventilated from an atmosphere impregnated with smoke and exhalations from sewers and graveyards, must be impure, and therefore injurious to health and life—is a proposition the truth of which cannot be doubted. To what degree it is injurious no one can possibly state numerically; the feat is simply impossible. We, therefore, think it unnecessary to follow Mr. Phillips through his long chain of statistical evidence on this head. We think the pith of the whole may be put into a nutshell. It simply amounts to this, that in those districts in which there is an excessive infantile mortality, fewer sickly children survive to die of scrofulous disease; that is, to die of scrofulous disease of the articulations, &c. So that, in fact, the greater the number of deaths from scrofula, the more healthy we may conclude the district to be. But this inference must be received with divers modifications.

*Influence of climate.* The belief that one climate is more favorable to life than another is universal, and is no doubt well founded, although no one has demonstrated (or, we believe, can demonstrate) the fact numerically. The salubrity of climates is relative. The adaptability of constitution to climate is an important element in the question. The Hindoo perishes prematurely in England; the European sickens or dies prematurely in India. Mr. Phillips can make out no facts which show that light, electricity, humidity of the atmosphere, or temperature, have any influence on the development of scrofula.

"When considering the prevalence of the disease, it was shown that we have no proof that climate, whether the temperature be high or low, variable or uniform, or the atmosphere be dry or humid, has any very obvious influence of itself, in producing or preventing scrofula. At St. Petersburg, with a mean temperature of 3·23, and a general mortality of 3·770; and Moscow, with a mean temperature of 3·6, and a general mortality of 4·010; and Iceland, where the Centigrade thermometer in winter indicates 20 minus—there appears to be less scrofula than at Lisbon, with its temperature of 71·2, or than at Amsterdam, Berlin, or Calcutta. So at Madeira, with its high mean temperature and low range, there is as much scrofula as among the juvenile convicts in Parkhurst prison. Other causes than climate must, then, in all these countries, exercise a most important influence in producing the disease; and among the causes of scrofula, we have seen that [innutritious] food holds the first place." (p. 221.)

*Employment as a cause of scrofula.* In considering this question, Mr. Phillips devotes his principal attention to the litigated question of the influence of factory labour in developing scrofula. He has secured several valuable and extensive statistical returns from the manufacturing districts; the substance of which we subjoin in his own words:

"In Leeds, Dr. T. Smith very kindly examined, at my request, 1095 children, employed in different factories, and examined 548 children of the same class, not employed in factories. The result is, that those not employed in factories exhibited marks of scrofula in 8 per cent. more instances than those whose days are spent in such establishments. In Manchester and other great manufacturing towns, a similar result has been obtained by examinations made at my request, and on so large a scale that there is every reason to feel confident in the opinion already expressed. Again, from his Dispensary practice, Dr. Smith has made me the following report:—'Of 916 persons, between seven and fourteen, the children of factory operatives, but not themselves employed in factories, 365, or 39 per cent., had enlarged glands, and 75, or 8 per cent., had scars resulting from scrofula. Of 567 persons, all under twenty-one, and employed in factories, 124, or 22 per cent., had scrofulous scars.'

"Mr. Poyser, the intelligent surgeon of Winksworth, kindly examined for me the people employed in Mr. Arkwright's mills, and the following are the results which he communicated:—'Persons examined, 798; total number having marks of scrofula, 29.'

....."I have obtained, through the kindness of Messrs. Horner and Saunders, the results of the examination of 6754 factory children, from which it appears that marks of scrofula were found in only 905 instances, or only 13½ per cent. The returns of Mr. Fereday, Mr. Davis, and that of other friends, who have kindly made a comparative examination of a large number of children, exhibit similar results; and they leave no doubt on my mind that children employed in factories are more free from scrofula than the average of children in England and Wales." (pp. 231-2.)

Dr. Martin furnished returns from the Mayfield Cotton Factory, situate in the parish of Clonegan, county of Waterford. The village is within a few hundred yards of the factory, and contains a population of 3075, of which 1061 are employed at the works. The following is a tabular result furnished by Dr. Martin as to the prevalence of scrofula amongst this population:

| | External population, 2015. | Factory operatives, 1061. |
|---|---|---|
| Hip disease | 6 | 2 |
| Knee ,, | 7 | 0 |
| Elbow ,, | 4 | 0 |
| Wrist ,, | 1 | 1 |
| Ankle ,, | 1 | 2 |
| Ulcers (scrofulous) | 15 | 2 |
| Phthisis | 10 | 8 |
| Ophthalmia | 7 | 4 |
| | 51 | 19 |

In investigating the evidence furnished by the registries of deaths as to the influence of factory towns, Mr. Phillips found that, in England and Wales, the deaths from scrofula, as compared with the total population, are as 7·6 to 100,000. In rural districts, the proportion is 9; in town districts it is 5. In factory towns, having a population of 2,043,038, the proportion is 4. In non-factory towns, having a population of 2,870,416, the proportion is 5 per 100,000. The alleged greater insalubrity of the employment in the woollen factory districts, as compared with the linen and cotton, so decidedly maintained by several authors, has not been confirmed by the inquiries Mr. Phillips has entered upon. In the linen and cotton districts, included in a table contained in the appendix, and having a population of nearly 1,000,000, the gross mortality is 2·786: that from consumption, ·477, or 1 in 209; that from scrofula, ·004, or 1 in 24,872. In the woollen districts, in the table, and comprising a population of nearly three quarters of a million, the gross mortality is 2·242: that from consumption, ·396; or 1 in 252; that from scrofula, ·005, or 1 in 17,877.

We do not think much reliance can be placed on these statistical deductions, although the data on which they are founded have no doubt been collected with great care and accuracy.

The question of the influence of factory labour on the sanitary condition and strength of the population divides itself into two main branches. First, how great is the influence exercised by it on the operatives? Secondly, how great is the influence exercised on their offspring? With regard to the first question, we may observe, that the proportionate number of persons actually working in factories having the marks of scrofula, or suffering from scrofulous disease, is no evidence of the influence of factory labour on the production of the disease, because the greatest sufferers would not be able to follow their work. A very large proportion of those engaged in this kind of labour might die in consequence of their occupation, and still the results of an inquiry like that instituted by Mr. Phillips would be favorable. We might go into a well-ordered factory, and say with Mr. Hamilton, "Gad! we find the cotton factories a specific for scrofula," if we extended our inquiries no farther, just as we might have reviewed the British Army on the Sutlej after the late battles, and say, "Gad, the battle has been a child's play, there's nobody seriously hurt!" Now as we would go to the military hospitals for the actual casualties of the campaign, so we should go to the homes of the sick workers, or trace them to the hospitals, or to the grave. The table supplied by Dr. Martin, of Mayfield, is defective in this point, it does not tell us how many of the "external population" had been employed at the works, or how long their period of service was; and particularly, it does not tell us how many of the persons suffering from scrofulous diseases in that population had been employed there, and for what period. It is certain that the injurious influence of factory labour is slow in its operation, and consequently an extended period of time should be one of the elements of the investigation.

With respect to the second question,—what is the influence of factory labour on the health and strength of the children of the operatives (we do not mean those children employed in the factories specifically), we may again observe, that these statistics are fallacious. The prevalence of scrofula amongst children under 5 years of age is not touched upon by Mr. Phillips's statistics. Further, the mortality of children under this age is excessive in the town districts as compared with the rural. According to Mr. Farr's tables, in Surrey, at the age of five, only 20 per cent. of those born at the same time have died, while in Liverpool the proportion is 47 per cent. Now it is not easy to state numerically how many of the children in manufacturing districts are cut off by tabes mesenterica, and other diseases of a scrofulous character, in early life, but there can be no doubt that the proportion is much greater than in the rural districts. It is also certain that the more numerous deaths during the earlier years will weed the population, as we have already shown, of many sickly lives, which under the more favorable circumstances of a country life would survive long enough to die of such diseases as would be entered as scrofula in the register. To give anything like trustworthy accuracy to statistical deductions as to the influence of factory labour on the health of the population in general, and on the development of any particular disease, it would be necessary to select a number of children the offspring of factory labourers; ascertain the deaths at each age; follow up the history of those who live long enough to be employed in the factories; note the diseases from which they suffer or die, and when arrived at puberty and married extend the inquiry to *their* offspring. Such an inquiry would, of course, involve a laborious and lengthened investigation, but we really think the known laws of hygiene forbid the cautious inquirer to draw positive conclusions as to the influence of factory labour from less extensive data.

Mr. Phillips's inquiries are certainly corroborative of the views we provisionally advocated a few years ago in our Fifteenth Volume, in opposition to the general prejudice then prevalent against the gigantic manufacturing system. Those prejudices, however, were, we believe, to a considerable degree justified, because the greater attention to the sanitary condition of their manufacturing population which characterizes many master manufacturers is only of comparatively recent development. Some of the new factories are splendid palaces, compared with the older places of labour. In short, the popular prejudice is founded on what was, and not exactly on what is. We would observe, however, that there are yet factories in which the modern hygienic appliances have not been so extensively adopted as in the newer structures; so that this inquiry to be rigidly accurate, should not be confined to the latter, but extend to all. It is certainly more convenient to make inquiries in the newly-built, large, and well-regulated factories, but it is precisely in those in which the evils of the factory system are most diligently obviated, and its advantages developed. Such an example, we presume, is the Mayfield works, which Dr. Martin assures us are very carefully regulated as to temperature and crowding.

*Treatment.* We now come to a more congenial, because a less controversial, subject. Mr. Phillips considers the treatment of scrofula under two heads, the prophylactic and the therapeutic. The prophylactic treatment insists upon well-assorted marriage as an important aid in preventing the occurrence of scrofula. Then the dietetics of infants should be regulated; they should be supplied plentifully with fresh air; and should be encouraged in all the active exercises in which young animals generally delight; and the games should be spontaneous, and left to the guidance of their own instincts. Mr. Phillips objects to infant schools as constraining young children in this respect. He observes:

> "I know that the health of those infants, who are suffered to amuse themselves as they please during the day is better, *cæteris paribus*, than that of those children who have been for many months regular attendants at infant schools. And the reason of the difference I apprehend to be this, that in children the blood is vigorously circulated through the entire frame, by means of the exertion of the muscular system, and this exertion of the muscular system can only be maintained by providing such amusement as will keep the body in motion. The listless walk around the school-rooms, though repeated many times a day, will not quicken the heart's action, and will not warm the hands and feet. And so long as the hands and feet, and the surface of the body remain cold for many hours of every day, so long the child will have congestion of some internal organs, and a state of permanent disease is readily induced; digestion is ill performed, nutrition is defective; and if this state of things be long continued, scrofula may be the consequence." (p. 251.)

We entirely agree with Mr. Phillips in these statements, and have always denounced infant schools conducted on the plan here referred to. But we believe, at the same time, that any plan involving such manifest disregard of all hygienic laws constitutes no necessary part of the system of infant schools. On the contrary, we believe that they may be made as directly conducive to bodily health, as to the regulation and improvement of the mind. And we are happy to know that some infant schools are so conducted.

We cordially agree with the following opinions, quoted from a communication by Mr. Turnbull, who reported on the Austrian School at Neustadt.

"Amid modern theories of education, and which prevail in other countries besides Germany, few perhaps are more particularly erroneous than the system which would be always teaching something; always in every form of play seeking to impart instruction. The gymnastics and equitations at Neustadt become thus as completely matters of study, and are probably performed with as much gravity of attention as the task of mathematics, or of history, because they are performed under the eye of the teacher. In the inaptitude of youth for any long continued application, nature herself points out the expediency of alternate repose to the mind, of entire vacancy of thought; but man too often endeavours to counteract this wise disposition by ever endeavouring to engage the attention by some new object of instruction. The animal spirits, those delightful harbingers of health and energy, mental and corporeal, are stunted in their very spring when the boy is debarred from those alternations of idle, thoughtless independence in his sports, which is not less essential to the formation of his future character than the practice of his severer studies. The mind is frittered away by the multitude of pursuits, and filled with a number of crude and confused ideas. It becomes paralysed by over-work, or precociously and morbidly active by over-excitement. A being of dull and orderly correctness may be produced by such discipline; or the memory may be overcharged (to the probable ruin of the reflecting power), so as to delight unthinking relatives with the multitude of acquired ideas: but as the lad was wanting the freshness of youth, so he will probably in after years be without the vigour of manhood." (p. 254.)

*Curative treatment of scrofula.* Mr. Phillips devotes several pages to the history of the cure of scrofula by the royal touch, in which much literary research is displayed. He thinks that the popular notion as to the remedial powers of the monarch were founded on *post hoc ergo propter hoc* evidence, the fallacy of which we have taken some pains to expose and denounce. Wiseman declares he was "a frequent eye-witness of many hundreds of cures performed by his Majesty's [Charles II] touch alone, without any assistance of chirurgery, and those many of them such as had tired out the endeavours of able chirurgeons before they came thither. It were endless to recite what I myself have seen," &c. Wiseman was a courtier; is it an unfair imputation on his character to add, that he knew his part well, and played it well? Mr. Phillips observes:

"The truth is, that of the scrofulous patients who suffer from enlarged glands in the neck, nine out of ten do get well under almost any rational plan of treatment. The tendency to improvement is most remarkable in spring and summer months those being the seasons of the year at which a large portion of the persons touched by Charles II were presented to his most gracious Majesty, and who, whether touched or not, might be reasonably expected to be much better by the end of summer. Ignorant as even many professional persons were at that time of the natural history of the disease, it was not surprising that they should attribute the cures which followed the touch to the ceremony itself. But with increased knowledge, we may form other judgments, and can, without presumption, refer those cures to other causes than the imposition of the king's hand." (p. 269.)

*Iodine.* Mr. Phillips thinks its virtues have been much overrated, and observes that the 35 cases reported by Lugol as cured out of 105 treated, would have been as well cured without it, if no other means than suitable food, air, clothing, and exercise, had been employed, and the season had been favorable. He further states :

"In my own practice I have exhibited every form of iodine extensively in cases of scrofula, and supposing the patient to remain exposed to the influence of the same conditions in which the disease was first manifested, and the period of the year to be that which has not been found favorable for the cure of the disease under other modes of treatment, I cannot say that I have reason to estimate the curative powers of iodine so highly as many others have done. I know that, among the out-patients of hospitals, whose circumstances remain unchanged, and who apply at the latter end of autumn or the beginning of winter, we may often exhibit iodine in every form for weeks or months without producing any sensible amelioration in the patient's condition. I know also that at the beginning of summer, a patient similarly affected and similarly treated, will, often in a few weeks, exhibit a marked improvement—but how much of this should be referred to iodine ? How much to season ?" (p. 275.)

Mr. Phillips, in common with all experienced practitioners, objects to the use of the simple tincture. He has found the iodide of iron a useful tonic, given in syrup, to the amount of four grains in the twenty-four hours. In whatever form the remedy be administered, he thinks it imprudent to continue its use longer than a fortnight, or three weeks, at a time. "At the end of that time the preparation should be set aside, aperient medicine should be employed, and its use should then be resumed with the same precautions. In this way, any virtues which the medicine possesses are more surely brought out, and the inconveniences sometimes experienced from its administration will be, as nearly as possible, avoided."

We have known numerous instances in which a scruple of the hydriodate of potass was taken daily for many weeks without any perceptible effect. The only rule we have for its discontinuance is the development of catarrhal symptoms, which in some people supervene on its administration even for a short period.

*Barium,* or rather the *chloride of barium* (the old muriate of baryta), is a remedy, in Mr. Phillips's opinion, little inferior to iodine :

"Barium, however," he observes, "seems to be a more certain stimulant than iodine, or, rather, we might say, irritant ; and, in my judgment, its use is clearly contraindicated where there is much free inflammatory excitability of the system. But in those cases where the tallow-like complexion, the pale tongue, and the languid circulation, accompanied by irritability of the mucous surfaces, are present, the virtues of the barium are often very remarkably demonstrated. I usually give it in solution, a grain to an ounce of distilled water, with ten drops of compound tincture of gentian. Of this solution, I commence with half an ounce twice a day, and on no occasion have I exceeded three grains in the day ; and up to this moment I have not experienced any check in the administration of the medicine." (p. 282.)

The *hydrochlorate of lime* is the basis of the anti-scrofulous nostrum of

Nieumann. Mr. Phillips has frequently used it in the following form: a drachm to twenty drachms of distilled water, of which a teaspoonful was taken in milk two or three times a day. He has carried the dose up to two teaspoonfuls, but has not exceeded that dose. He is not convinced that it has any very evident action on scrofulous glands, but in moderate doses it is more generally tolerated than the chloride of barium.

The alkalis are of doubtful value; so also burnt sponge.

*Cod-liver oil* is useful, Mr. Phillips thinks, rather as an animal oil, than because it contains infinitesimal doses of iodine or bromine:

"The conviction on my mind is, that when good is derived from it, it is to be referred to the effect of the animal oil in improving digestion and nutrition, rather than to the presence of iodine; and if Popkins's impression be correct, that he has observed quite as much good to follow the daily use of fried bacon in such cases, and if it be further true, which I by no means admit, that butchers, oilmen, tallow-chandlers, tanners, and other persons who are continually coming in contact with fatty matter, are particularly robust and well nourished, and are known to be remarkably free from scrofula, then the case in favour of the oily principle is so much the stronger. If the impression be correct, that cases are occasionally presented in which the good effects of cod-liver oil are remarkably apparent, and if the amelioration seem to concur with a much improved condition as to nutrition, I conceive myself justified in assuming that the one is the consequence of the other without being required to frame a theory why animal oil improves nutrition." (p. 288.)

*Sea water and sea bathing.* Mr. Phillips is full of doubts as to the merits of these remedies. Change of inland air and cold bathing may be equally, if not more, useful. Some years ago, an experiment as to the value of sea air was made in nine obstinate cases of scrofula, in the St. Marylebone Infirmary. They were sent to Margate, and remained there several months.

"On their return their condition was carefully ascertained. The glandular tumors had very nearly, but not completely subsided; of the sinuses one was dried up, the other nearly so. In the former two cases of joint affection no amelioration was experienced in the local disease, but the general health was improved; the third patient, after remaining at Margate some weeks, came home to die.

"The eight surviving children had returned home at the close of the Margate season. In the succeeding November, three of them were again under treatment: one with glandular tumor, which had re-appeared; one with a re-opened ulcer; the other with a single sinus, still discharging. These results are, I conceive, in perfect accordance with the experience of careful observers; and certainly they do not tend to support the prejudice which exists in favour of the superior efficacy of sea-side residence in cases of scrofula. That those ganglia in which scrofulous matter had been deposited should continue foci of irritation is natural, because I know no satisfactory evidence that scrofulous deposits are ever absorbed. They, therefore, continue to exist, and, like any other foreign body, may excite new irritation from very slight causes. That a sinus should heal when properly treated, provided all the scrofulous deposit be removed, is to be expected; but whether the sea-side did much to determine the subsidence of irritation around the gland in one set of the cases above noticed, or to heal the fistula in the other, is with me a matter of doubt." (p. 293.)

*Mineral waters* are in as little estimation with Mr. Phillips as sea bathing. We subjoin some therapeutic statistics of French mineral waters used for the cure of scrofula, together with Mr. Phillips's remarks. The facts are derived from the reports of the inspectors of mineral waters in France:

"SCROFULOUS ENLARGEMENTS.

| | Cases observed. | Cures. | Ameliorations. | No benefit. | Deaths. |
|---|---|---|---|---|---|
| Bourbonne . . | 29 | 2 | 16 | 11 | 0 |
| Balaruc . . | 46 | 14 | 16 | 14 | 2 |
| „ . . | 13 | 0 | 5 | 8 | 0 |
| Mont d'Or . . | 19 | 3 | 5 | 11 | 0 |
| Néris . . | 4 | 0 | 2 | 2 | 0 |
| Bagnoles (Lozère) . | 78 | 17 | 38 | 23 | 0 |
| Bagnères de Luchon . | 41 | 14 | 10 | 17 | 0 |
| | 230 | 50 | 92 | 86 | 2 |

ABSCESSES, ULCERS, FISTULÆ.

| | Cases observed. | Cures. | Ameliorations. | No benefit. | Deaths. |
|---|---|---|---|---|---|
| Bourbonne . . | 132 | 57 | 62 | 13 | 0 |
| Bourbon l'Archambaut | 43 | 18 | 15 | 10 | 0 |
| | 175 | 75 | 77 | 23 | 0 |

"The above table implies, I believe, as favorable an estimate as can properly be made of the influence of mineral waters in the cure of scrofula, and certainly the result does not show that they exercise any very decided curative influence over the disease.

"That they have been more indebted for the credit they possess to the enthusiasm of friends, than to the faithful register of the cures which it is alleged have resulted from their employment, is, I think, true. And no doubt M. Patissier was near the truth when he said, 'Les eaux minérales naturelles guérissent quelquefois, soulagent et consolent toujours.'" (pp. 299, 300.)

The season of the year has, in Mr. Phillips's opinion, much influence in the development and amelioration of scrofula:

"I have fully satisfied myself that scrofulous cases are most numerous and most aggravated in spring and the beginning of summer; that they are least frequent and most ameliorated at the commencement or the middle of autumn; and that at the one period they have been aggravated by the cold of winter, at the other they have been ameliorated by the warmth of summer; and this has happened when all other influences have been apparently unchanged.

"When do we send patients tn the sea-side? Precisely at that season when they would improve anywhere. When do we find any remedies best succeed? Precisely when the season is becoming favorable. When do we find all medicinal agents comparatively powerless? Precisely when we get no help from the season. True it is, we may take a poor child from the streets in the depth of winter, and give him good food and lodging, and his disease will be improved, and this without reference to the season; but then we have improved nutrition. But let a poor child remain at home, and we shall usually find every specific fail to improve his condition until favoured by season; and let almost any one of the unsuccessful remedies be used when the end of spring comes, and its apparent good effects will soon be obvious enough. Now, if that position be correct, it must be evident how defective the estimate of every plan of treatment is which does not include the influence of season." (p. 301.)

The *Appendix* contains many of the returns *in extenso*, which Mr. Phillips so industriously obtained from a numerous and wide circle of correspondents. One of the first of these documents is a short essay by Professor Albers, on the difference between scrofula and tubercles—the latter term being used to signify not only tubercles of the lungs, but also phthisis. In looking over this we could not avoid remarking how completely Professor Albers had mistaken the point at issue. The differences which he lays down are simply differences in semeiology, and not in pathology—differences which arise from a difference in the organ affected, and not from a difference in the nature of the two diseases. The symptoms of disease of the lymphatic glands and of the lungs must necessarily differ; the one is a "noble organ," intimately connected with vital action, the other of secondary importance.

In concluding our review of Mr. Phillips's work, we feel we cannot estimate too highly the industry and perseverance with which he has pursued his researches into so important a subject, and we think the mass of valuable facts he has collected gives such a value to his work as will secure it a place amongst the important class of "books of reference." We trust Mr. Phillips will not only continue his researches, but will also adopt a still more comprehensive plan. He should, we think, carefully avoid narrowing in any way the field of his inquiry. Scrofula should be investigated as "a disease of the constitution." It should be traced step by step through each class of organs, and as well in their histological constitution as in their successive development from infancy to old age. The prosecution of the inquiry on this philosophical basis may raise a monument to his fame, which shall match if not overshadow the labours of Louis on a kindred subject, and possibly merge them into a higher generalization. The scrofulous diseases of the glandular structures of infancy might form one group; of youth another; of puberty another; of middle age another; of old age another. And although the main inquiry should be directed to the glandular and parenchymatous organs, the cutaneous cellular tissue, and the structures imbedded in the epidermis should not be forgotten. There would thus be a large family group of diseases investigated in their general and mutual relations, comprising not only mesenteric disease, scrofulous diarrhœa, enlarged submental and cervical glands, and scrofulous articulations, but also scrofulous disease of the gastro-enteric mucous membrane and its diverticula into the Eustachian tube, salivary glands, liver, kidneys, &c. (constituting obscure forms of indigestion, &c.); scrofulous hydrencephalus, hypertrophy of the lips, alæ nasi, and general subcutaneous tissue; scrofulous ophthalmia, cutaneous eruptions, abscesses, &c. We are convinced that anything short of a comprehensive and philosophical inquiry like this—of which the above is a meagre outline—will be insufficient to determine the litigated questions connected with the pathology and relations of scrofula, or materially advance the treatment of tubercular diseases in general.

